# Supplementary material for: Arabidopsis LSH10 transcription factor and OTLD1 histone deubiquitinase interact and transcriptionally regulate the same target genes
Source: Commun Biol. 2023 Jan 17;6:58. doi: 10.1038/s42003-023-04424-x (PMC9845307; doi:10.1038/s42003-023-04424-x)
Supplement: Supplementary file 2 — Supplementary Material [file 42003_2023_4424_MOESM2_ESM.pdf]

Supplementary Information

Supplementary Figure 1. Identification of the *LSH10* clone by screening the Arabidopsis yeast two-hybrid library with a truncated OTLD1 bait

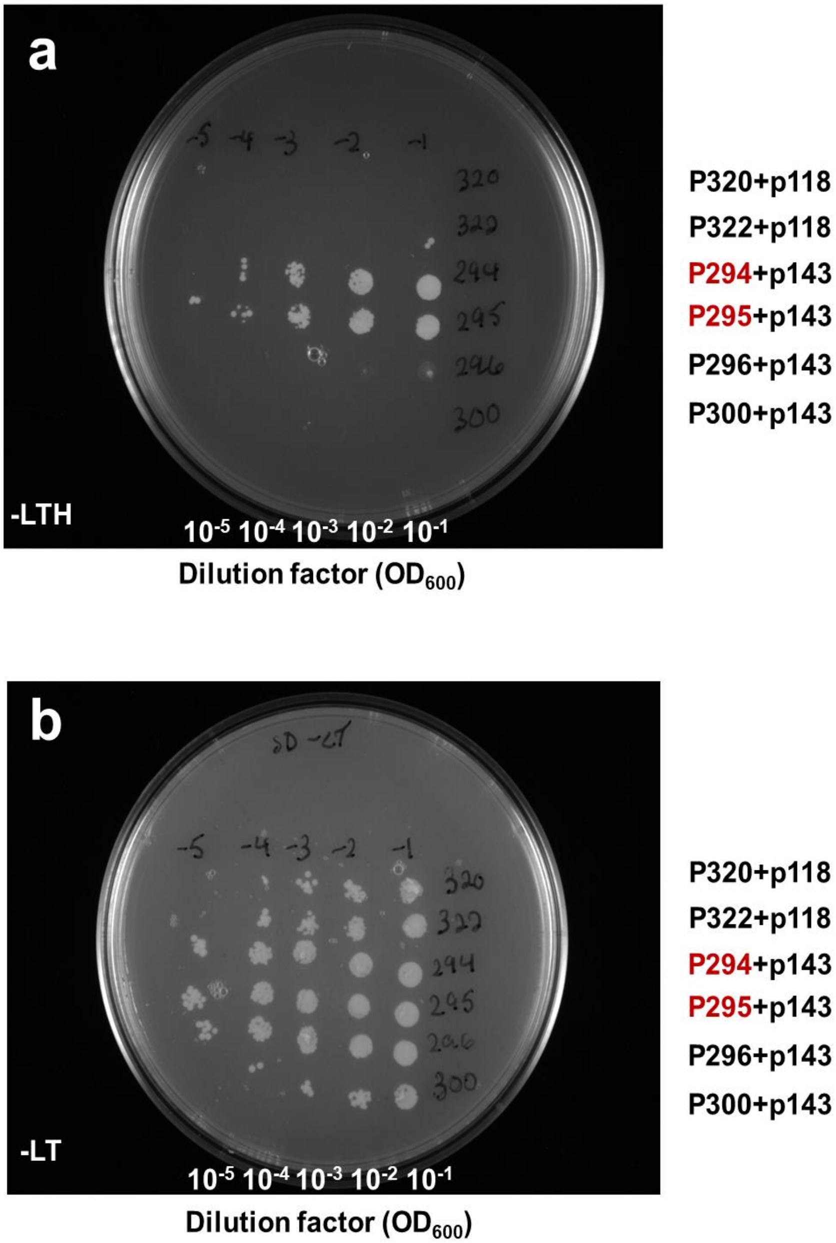

The screen was performed using the yeast strain L40 (1), co-transformed with pSTT91- and pGAD424-derived constructs expressing the bait (amino acid residues 1-223 of 506; for PCR primers, used to generate this bait, see Supplementary Data 1) and the library tagged with LexA and Gal4 AD, respectively. The cells were plated at the indicated dilutions on the selective leucine, tryptophan, and histidine-deficient medium (-LTH) (**a**) or on the non-selective leucine and tryptophan-deficient medium (-LT) (**b**). Cell growth was recorded after incubation for 2-3 days at 28°C. Protein interaction is detected as cell growth on the LTH medium. Cell viability is indicated by growth on the LT medium. The following baits and prays were coexpressed in the indicated combinations: p143, the truncated OTLD1; p118, lamin C (1, 2); P294 and P295, independent LSH10 clones (highlighted in red font), P296, peroxidase superfamily protein PRXR1; P300, P320, and P322, unidentified randomly picked clones from the two-hybrid library.

Supplementary Figure 2. Structure of LSH10 (ALOG domain)

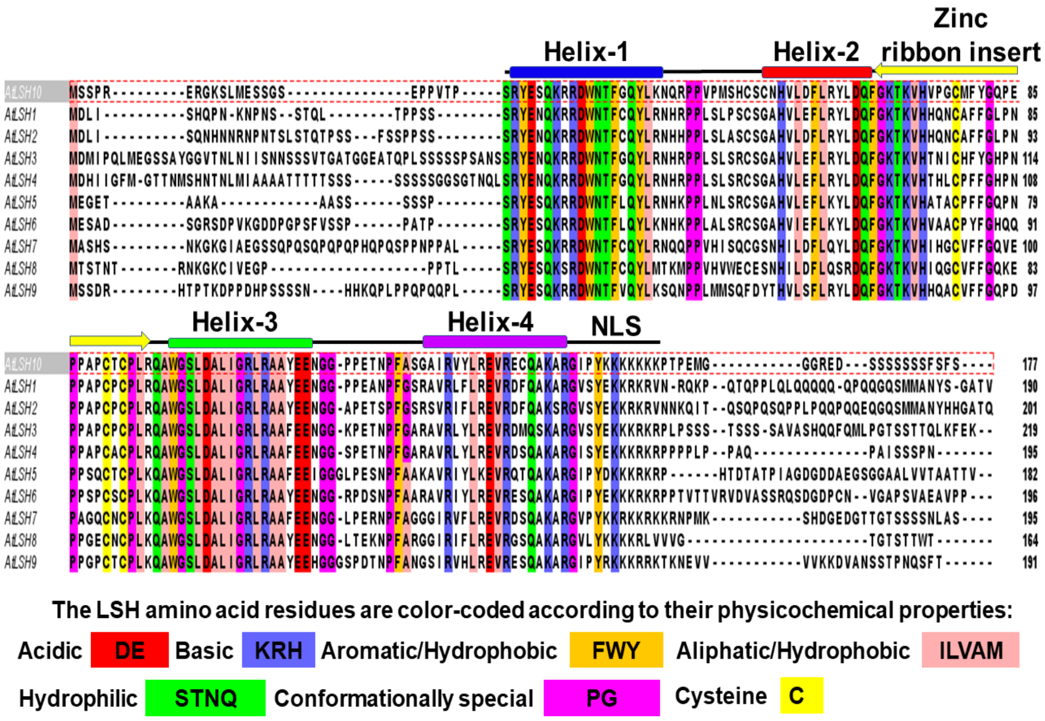

Sequence alignment of the nine Arabidopsis LSH (AtLSH) protein family members and conserved features of their ALOG domain. Sequences are color-coded based on their conservation at 100% consensus. The coloring scheme and the conserved amino acids are indicated below the alignment. The highly conserved ALOG domain includes a zinc ribbon insert structure, four helices, and an NLS.

Supplementary Figure 3. Evolutionary analysis of the LSH protein in plants

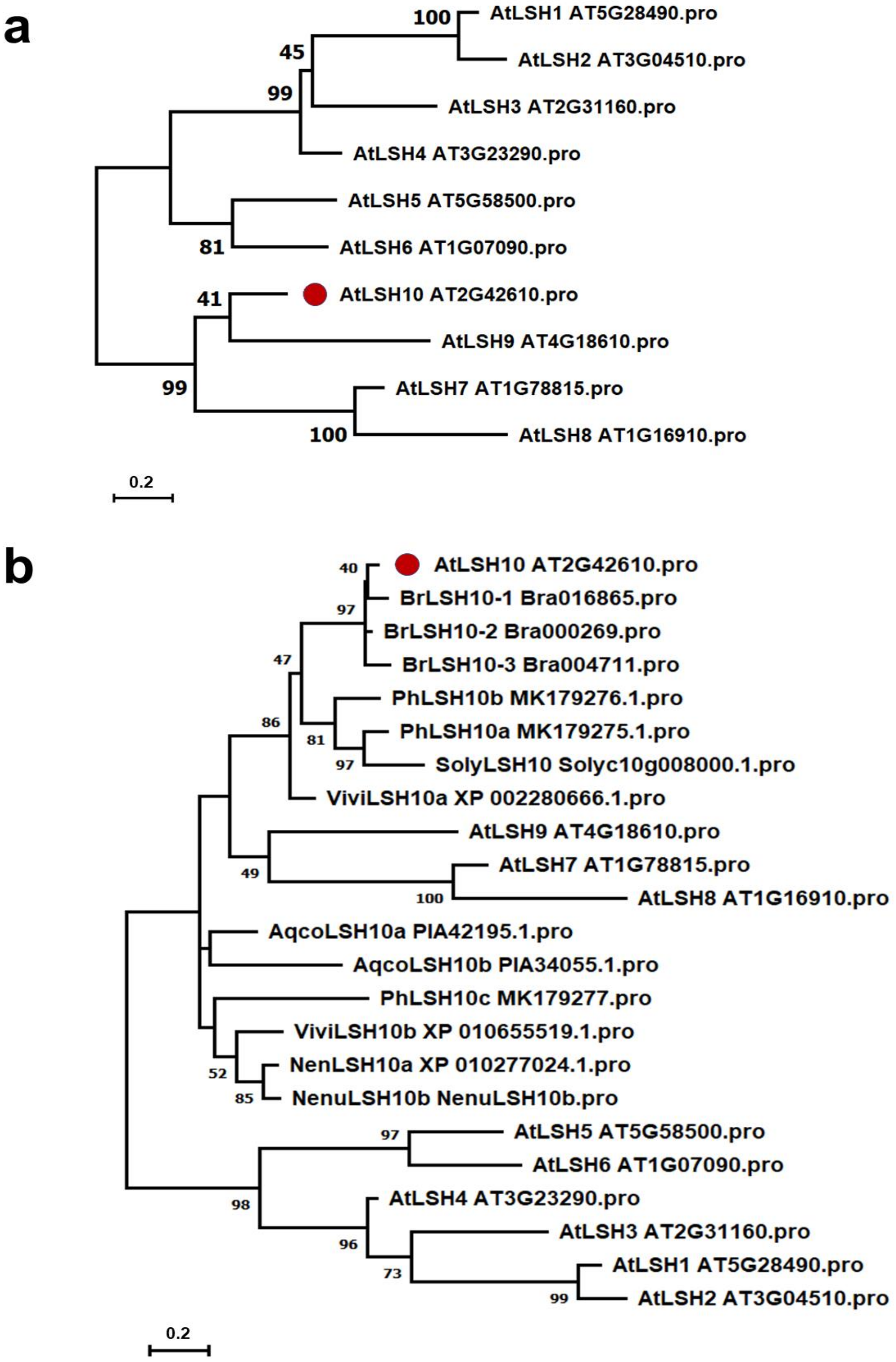

(**a**) Arabidopsis LSH proteins (AtLSH). (**b**) LSH proteins in diverse dicotyledonous plant species. The evolutionary history of AtLSHs was inferred using the Maximum Likelihood method and JTT matrix-based model (3). The trees with the highest log likelihood (-2787.03 for panel A and -4185.81 for panel B) are shown. The percentage of trees in which the associated taxa clustered together is shown next to the branches. Initial tree(s) for the heuristic search were obtained automatically by applying Neighbor-Join and BioNJ algorithms to a matrix of pairwise distances estimated using the JTT model, and then selecting the topology with a superior log likelihood value. A discrete Gamma distribution was used to model evolutionary rate differences among sites [5 categories (+G, parameter = 0.5059) for panel A and (+G, parameter = 0.5524) for panel B]. The trees are drawn to scale, with branch lengths measured in the number of substitutions per site. These analyses involved 10 and 23 amino acid sequences for panels **a** and **b**, respectively. There was a total of 225 and 226 positions in the final dataset for panels **a** and **b**, respectively. Scale bars, 0.2 amino acid substitutions per site. The individual sequences are identified by the name of the protein and the corresponding AGI locus code (for Arabidopsis proteins) or Gene ID (for other plant proteins); LSH10 is highlighted with a red dot. Evolutionary analyses were conducted in MEGA X (4). The plant species abbreviations are: At, *Arabidopsis thaliana;* Br, *Brassica rapa;* Ph, *Petunia hybrida;* Soly, *Solanum lycopersicum;* Vivi, *Vitis vinifera;* Aqco*, Aquilegia coerulea;* Nenu, *Nelumbo nucifera.*

Supplementary Figure 4. Alignment of the predicted secondary structure of LSH10 and the N-terminal DNA binding domain of the Cre recombinase

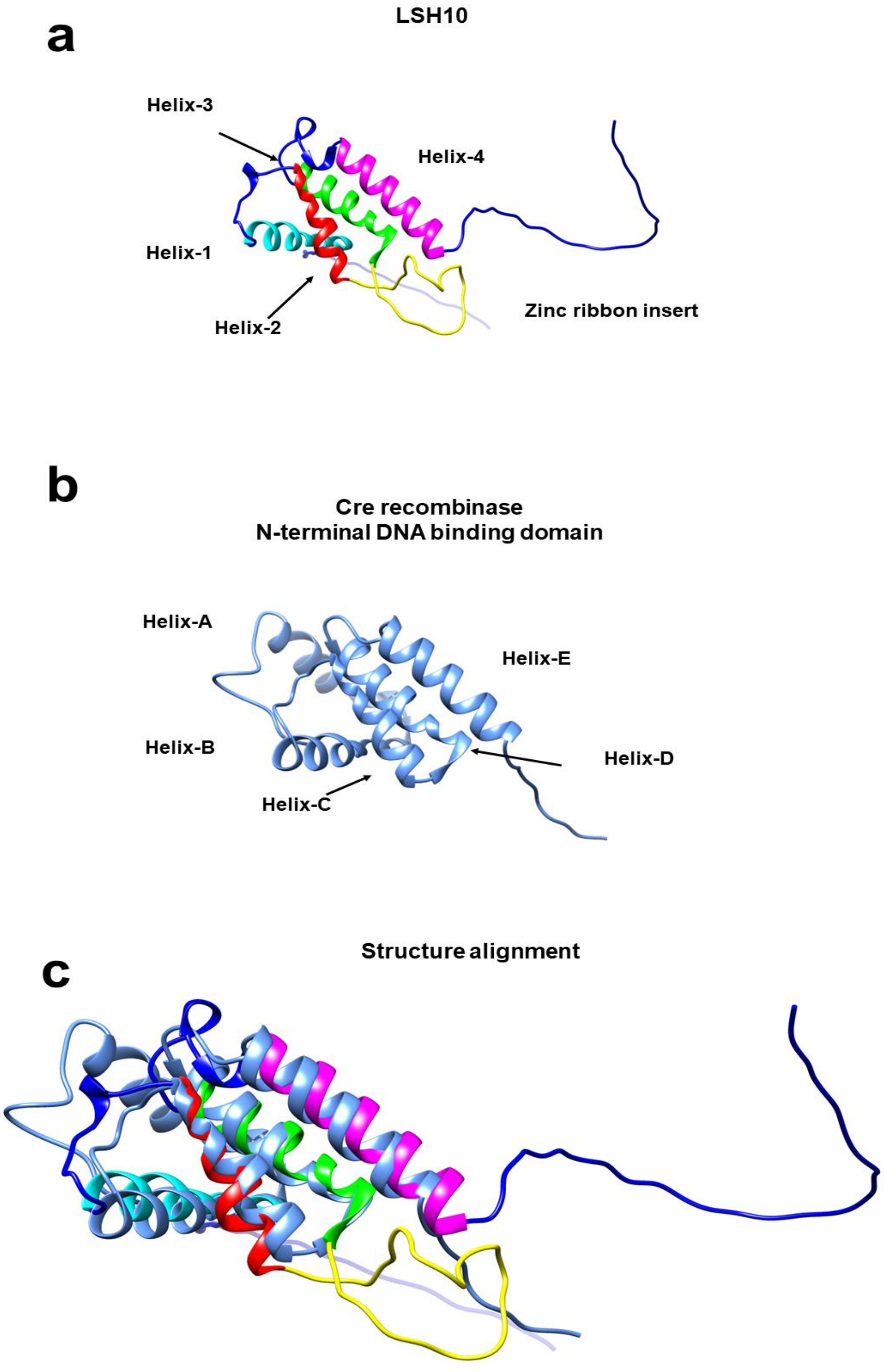

(**a**) The ribbon diagram of LSH10 predicted by AlphaFold (AF-Q9S7R3). Helices 1, 2, 3, 4, and zinc ribbon insert conserved in the ALOG domain are colored in cyan, red, green, magenta, and yellow, respectively. (**b**) The ribbon diagram of the Cre recombinase N-terminal DNA binding domain (PDB: 1ouqA) with its helices A-E is colored in cornflower blue. (**c**) Structure alignment between LSH10 and the Cre recombinase N-terminal DNA binding domain. Helices 1, 2, 3, and 4 of LSH10 align with helices B, C, D, and E of the Cre recombinase, respectively.

Supplementary Figure 5. Representative confocal images of cells expressing each of the donor and the acceptor constructs separately

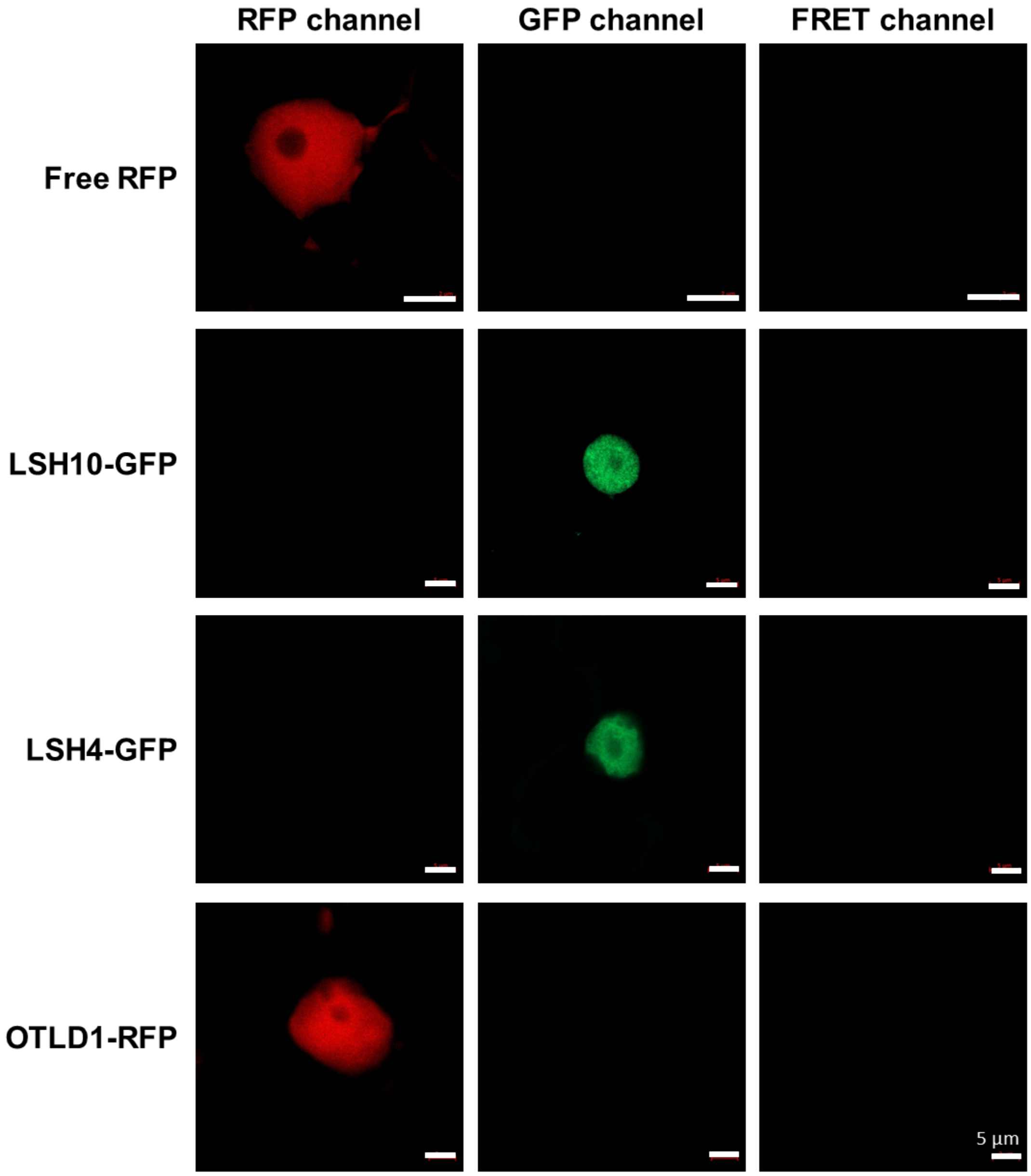

The images were obtained as described in Methods and utilized by the ImageJ plug-in PixFRET software for deriving the bleed-through values for SE-FRET. Scale bars: 5 µm.

Supplementary Figure 6. Uncropped images of the OSR2 and WUS EMSA gels shown in Fig. 5b

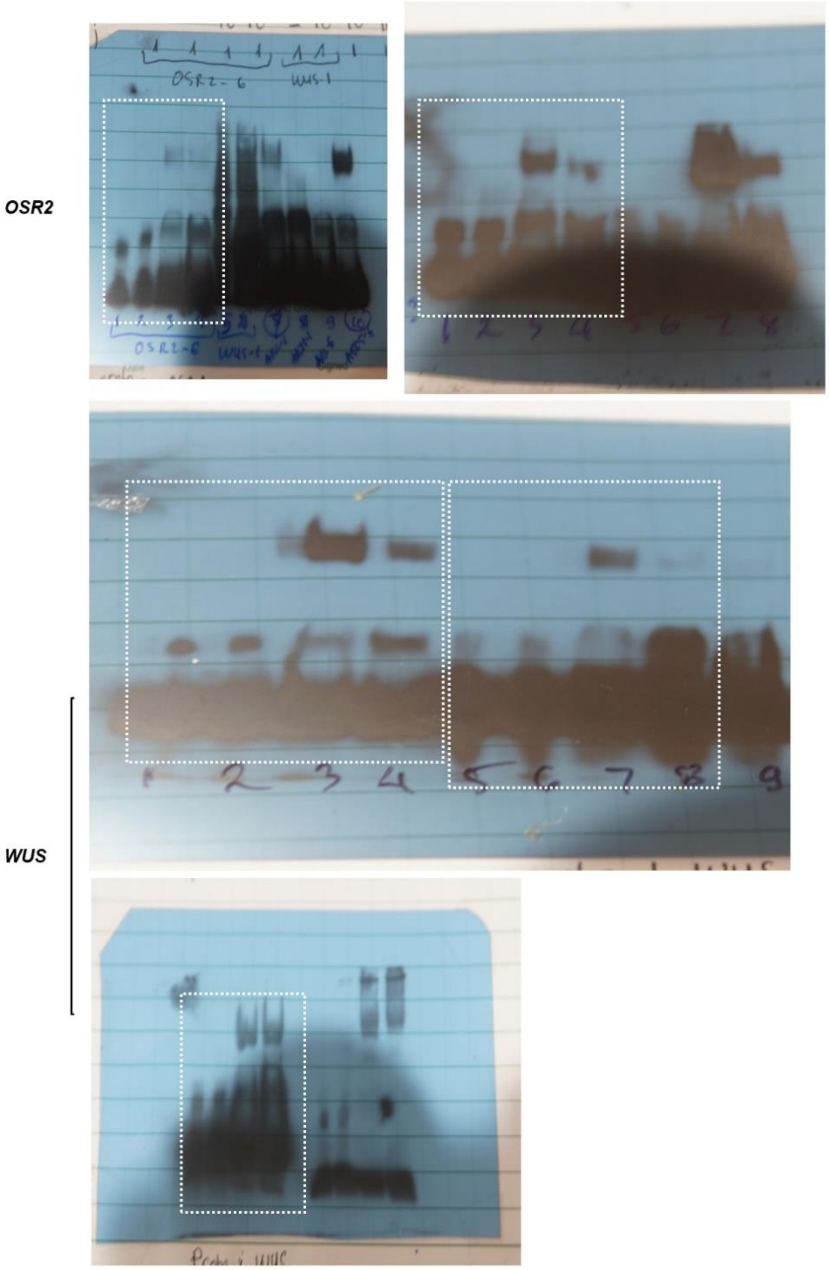

The images were obtained as described in Methods. Dotted rectangles indicate the parts of the images used in the corresponding panels in Fig. 5b.

Supplementary Figure 7. Uncropped images of the ABI5 and ARL EMSA gels shown in Fig. 5b

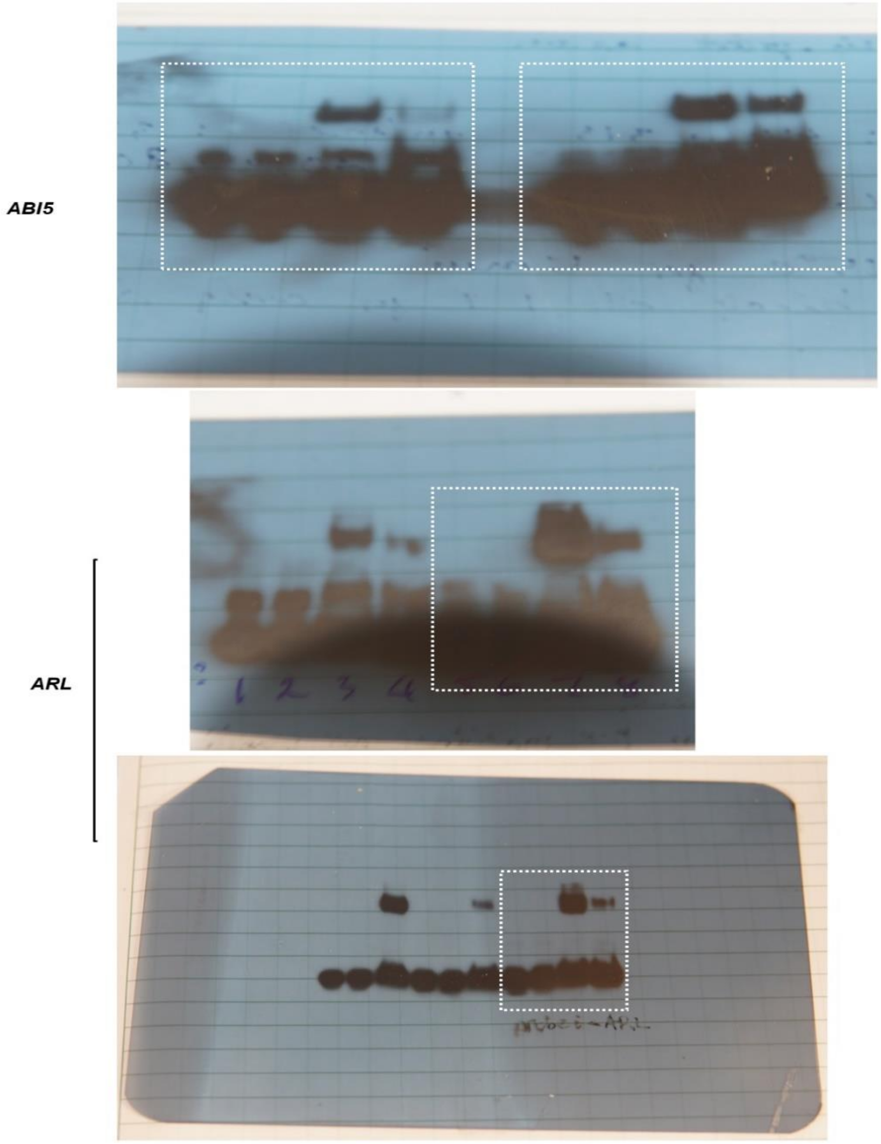

The images were obtained as described in Methods. Dotted rectangles indicate the parts of the images used in the corresponding panels in Fig. 5B.

Supplementary Figure 8. Uncropped images of the EMSA gels for negative probes shown in Fig. 5b

ARL, OSR2, WUS

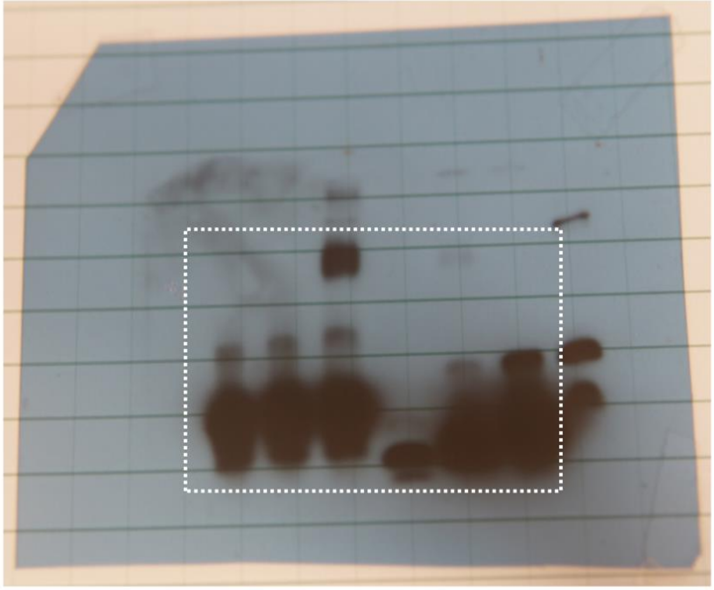

The images were obtained as described in Methods. Dotted rectangles indicate the parts of the images used in the corresponding panels in Fig. 5B.

Supplementary References

1. Hollenberg, S.M., Sternglanz, R., Cheng, P.F., and Weintraub, H. (1995) Identification of a new family of tissue-specific basic helix-loop-helix proteins with a two-hybrid system. *Mol. Cell. Biol.* **15**, 3813-3822.

2. Bartel, P., Chien, C., Sternglanz, R., and Fields, S. (1993) Elimination of false positives that arise in using the two-hybrid system. *Biotechniques* **14**, 920-924.

3. Jones, D.T., Taylor, W.R., and Thornton, J.M. (1992) The rapid generation of mutation data matrices from protein sequences. *Comput. Appl. Biosci.* **8**, 275-282.

4. Kumar, S., Stecher, G., Li, M., Knyaz, C., and Tamura, K. (2018) MEGA X: molecular evolutionary genetics analysis across computing platforms. *Mol. Biol. Evol.* **35**, 1547-1549.
