# Supplementary material for: Arabidopsis LSH10 transcription factor and OTLD1 histone deubiquitinase interact and transcriptionally regulate the same target genes
Source: Commun Biol. 2023 Jan 17;6:58. doi: 10.1038/s42003-023-04424-x (PMC9845307; doi:10.1038/s42003-023-04424-x)
Supplement: Supplementary file 3 — Description of Additional Supplementary Files [file 42003_2023_4424_MOESM3_ESM.pdf]

Description of Additional Supplementary Files

File name: Supplementary Data 1

Description: Primers used for DNA amplification, molecular cloning, and detection.

File name: Supplementary Data 2

Description: Raw data for quantification of AB-FRET (for Fig. 2C).

File name: Supplementary Data 3

Description: Raw data for quantification of the coexpression of the LSH10 and OTLD1 genes in different organs of the wild-type Arabidopsis plants (for Fig. 3).

File name: Supplementary Data 4

Description: Raw data for quantification of the increase in expression of the target genes in the lsh10-1, and lsh10-2 plants (for Fig. 4B,C). WT, wild-type.

File name: Supplementary Data 5

Description: Raw data for quantification of the transcriptional repression of the target genes in the lsh10-1/LSH10-His6 plants (for Fig. 4D). WT, wild-type.

File name: Supplementary Data 6

Description: Raw data for quantification of the qChIP analysis of the association of LSH10-His6 with the chromatin of the target genes (for Fig. 6A). WT, wild-type.

File name: Supplementary Data 7

Description: Raw data for quantification of the qChIP analysis of the increase in H2B monoubiquitylation of the target chromatin (for Fig. 6B). WT, wild-type.
