# Supplementary material for: Arabidopsis LSH10 transcription factor and OTLD1 histone deubiquitinase interact and transcriptionally regulate the same target genes
Source: Commun Biol. 2023 Jan 17;6:58. doi: 10.1038/s42003-023-04424-x (PMC9845307; doi:10.1038/s42003-023-04424-x)
Supplement: Supplementary file 5 — NR reporting summary [file 42003_2023_4424_MOESM5_ESM.pdf]

nature portfolio

Corresponding author(s): Mi Sa Vo Phan

Last updated by author(s): 2023/01/02

Reporting Summary

Nature Portfolio wishes to improve the reproducibility of the work that we publish. This form provides structure for consistency and transparency in reporting. For further information on Nature Portfolio policies, see our [Editorial Policies] and the [Editorial Policy Checklist].

Statistics

For all statistical analyses, confirm that the following items are present in the figure legend, table legend, main text, or Methods section.

n/a	Confirmed	
☐	☒	The exact sample size (*n*) for each experimental group/condition, given as a discrete number and unit of measurement
☐	☒	A statement on whether measurements were taken from distinct samples or whether the same sample was measured repeatedly
☐	☒	The statistical test(s) used AND whether they are one- or two-sided<br>*Only common tests should be described solely by name; describe more complex techniques in the Methods section.*
☒	☐	A description of all covariates tested
☒	☐	A description of any assumptions or corrections, such as tests of normality and adjustment for multiple comparisons
☒	☐	A full description of the statistical parameters including central tendency (e.g. means) or other basic estimates (e.g. regression coefficient) AND variation (e.g. standard deviation) or associated estimates of uncertainty (e.g. confidence intervals)
☐	☒	For null hypothesis testing, the test statistic (e.g. *F*, *t*, *r*) with confidence intervals, effect sizes, degrees of freedom and *P* value noted<br>*Give P values as exact values whenever suitable.*
☒	☐	For Bayesian analysis, information on the choice of priors and Markov chain Monte Carlo settings
☒	☐	For hierarchical and complex designs, identification of the appropriate level for tests and full reporting of outcomes
☒	☐	Estimates of effect sizes (e.g. Cohen's *d*, Pearson's *r*), indicating how they were calculated

Our web collection on [statistics for biologists] contains articles on many of the points above.

Software and code

Policy information about [availability of computer code]

Data collection	Zen 3.0 (system) was used to collect images from confocal fluorescent microscope (LSM 900) as TIF format. ImageJ and ImageJ plug-in PixFRET software were used for analyses of FRET images. For qPCR, Ct value were exported from tQuantStudio™ 3 Real-Time PCR System 377 (Applied Biosystems by Thermo Fisher Scientific) as excel format. DRNApred, DISPLAR, DP-Bind were used to predict DNA contacting residues of AtLSH10. HDOCK website was used to predict the DNA-binding mode of AtLSH10. MEGA X software (Maximum Likelihood method and JTT matrix-based model) was used to generate phylogenetic tree. AlphaFold and Chimera 1.14 was used to predict and generate secondary structure of LSH10. Multiple Expectation Maximizations for Motif Elicitation (MEME) online tool was used to search for conserved motifs on promoters.
Data analysis	Two-tailed t-test, using Microsoft excel

For manuscripts utilizing custom algorithms or software that are central to the research but not yet described in published literature, software must be made available to editors and reviewers. We strongly encourage code deposition in a community repository (e.g. GitHub). See the Nature Portfolio [guidelines for submitting code & software] for further information.

Data

Policy information about availability of data

All manuscripts must include a data availability statement. This statement should provide the following information, where applicable:

- Accession codes, unique identifiers, or web links for publicly available datasets
- A description of any restrictions on data availability
- For clinical datasets or third party data, please ensure that the statement adheres to our policy

Figure 2C, Figure 3, Figure 4B, Figure 4C, Figure 4D, Figure 6A, Figure 6B.

Human research participants

Policy information about studies involving human research participants and Sex and Gender in Research.

Reporting on sex and gender	*Use the terms sex (biological attribute) and gender (shaped by social and cultural circumstances) carefully in order to avoid confusing both terms. Indicate if findings apply to only one sex or gender; describe whether sex and gender were considered in study design whether sex and/or gender was determined based on self-reporting or assigned and methods used. Provide in the source data disaggregated sex and gender data where this information has been collected, and consent has been obtained for sharing of individual-level data; provide overall numbers in this Reporting Summary. Please state if this information has not been collected. Report sex- and gender-based analyses where performed, justify reasons for lack of sex- and gender-based analysis.*
Population characteristics	*Describe the covariate-relevant population characteristics of the human research participants (e.g. age, genotypic information, past and current diagnosis and treatment categories). If you filled out the behavioural & social sciences study design questions and have nothing to add here, write "See above."*
Recruitment	*Describe how participants were recruited. Outline any potential self-selection bias or other biases that may be present and how these are likely to impact results.*
Ethics oversight	*Identify the organization(s) that approved the study protocol.*

Note that full information on the approval of the study protocol must also be provided in the manuscript.

Field-specific reporting

Please select the one below that is the best fit for your research. If you are not sure, read the appropriate sections before making your selection.

☒ Life sciences ☐ Behavioural & social sciences ☐ Ecological, evolutionary & environmental sciences

For a reference copy of the document with all sections, see nature.com/documents/nr-reporting-summary-flat.pdf

Life sciences study design

All studies must disclose on these points even when the disclosure is negative.

Sample size	No samples size was performed. The sample size were experimentally selected based on the variation of mean. For examples, for quantification of AB-FRET, n = 13. For the qPCR experiments (for quantification of the increase in expression and the transcriptional repression of the target genes, n = 8; for quantification of the coexpression of the LSH10 and OTLD1 genes in different organs of the wild-type Arabidopsis plants: flowers, roots, stems, n=7; cauline leaves, rosette leaves, n=8). For quantification of the qChIP analysis of the association of LSH10-His6 with the chromatin of the target genes: Wild type, n = 5, Transgenic plants, n = 7. For quantification of the qChIP analysis of the increase in H2B monoubiquitylation of the target chromatin, Wild type, n = 7, transgenic plants, n > 3.
Data exclusions	No exclusion
Replication	3 times. All replications are consistent
Randomization	The wildtype, mutant, and complementation plants were grown in same culture condition. Tissue collection and measurement were conducted in same conditions.
Blinding	Blinding is not available

Reporting for specific materials, systems and methods

We require information from authors about some types of materials, experimental systems and methods used in many studies. Here, indicate whether each material, system or method listed is relevant to your study. If you are not sure if a list item applies to your research, read the appropriate section before selecting a response.

Materials & experimental systems

n/a	Involved in the study
☐	☒ Antibodies
☒	☐ Eukaryotic cell lines
☒	☐ Palaeontology and archaeology
☒	☐ Animals and other organisms
☒	☐ Clinical data
☒	☐ Dual use research of concern

Methods

n/a	Involved in the study
☒	☐ ChIP-seq
☒	☐ Flow cytometry
☒	☐ MRI-based neuroimaging

Antibodies

Antibodies used	Non- specific rabbit IgG Isotype Control (Invitrogen, WB317638), rabbit His-tag antibody (GenScript, A00174-40), anti-monoubiquityl-histone H2B (Lys-120) (5546S, Cell Signaling Technology, Inc.).
Validation	No validation
